# Oligo-painting and GISH reveal meiotic chromosome biases and increased meiotic stability in synthetic allotetraploid *Cucumis* ×*hytivus* with dysploid parental karyotypes

**DOI:** 10.1186/s12870-019-2060-z

**Published:** 2019-11-06

**Authors:** Qinzheng Zhao, Yunzhu Wang, Yunfei Bi, Yufei Zhai, Xiaqing Yu, Chunyan Cheng, Panqiao Wang, Ji Li, Qunfeng Lou, Jinfeng Chen

**Affiliations:** 1 State Key Laboratory of Crop Genetics and Germplasm Enhancement, College of Horticulture, Nanjing Agricultural University, Weigang Street No.1, Nanjing, 210095 China; 2 Institue of Horticulture, Zhejiang Academy of Agriculture Sciences, Hangzhou, 310021 China

**Keywords:** Allopolyploid, Interspecies hybridization, Meiotic instability, Karyotype variation, Oligo-FISH

## Abstract

**Background:**

Meiosis of newly formed allopolyploids frequently encounter perturbations induced by the merging of divergent and hybridizable genomes. However, to date, the meiotic properties of allopolyploids with dysploid parental karyotypes have not been studied in detail. The allotetraploid *Cucumis* ×*hytivus* (HHCC, 2n = 38) was obtained from interspecific hybridization between *C. sativus* (CC, 2n = 14) and *C. hystrix* (HH, 2n = 24) followed by chromosome doubling. The results of this study thus offer an excellent opportunity to explore the meiotic properties of allopolyploids with dysploid parental karyotypes.

**Results:**

In this report, we describe the meiotic properties of five chromosomes (C5, C7, H1, H9 and H10) and two genomes in interspecific hybrids and *C.* ×*hytivus* (the 4th and 14th inbred family) through oligo-painting and genomic in situ hybridization (GISH). We show that 1) only two translocations carrying C5-oligo signals were detected on the chromosomes C2 and C4 of one 14th individual by the karyotyping of eight 4th and 36 14th plants based on C5- and C7-oligo painting, and possible cytological evidence was observed in meiosis of the 4th generation; 2) individual chromosome have biases for homoeologous pairing and univalent formation in F_1_ hybrids and allotetraploids; 3) extensive H-chromosome autosyndetic pairings (e.g., H-H, 25.5% PMCs) were observed in interspecific F_1_ hybrid, whereas no C-chromosome autosyndetic pairings were observed (e.g. C-C); 4) the meiotic properties of two subgenomes have significant biases in allotetraploids: H-subgenome exhibits higher univalent and chromosome lagging frequencies than C-subgenome; and 5) increased meiotic stability in the S_14_ generation compared with the S_4_ generation, including synchronous meiosis behavior, reduced incidents of univalent and chromosome lagging.

**Conclusions:**

These results suggest that the meiotic behavior of two subgenomes has dramatic biases in response to interspecific hybridization and allopolyploidization, and the meiotic behavior harmony of subgenomes is a key subject of meiosis evolution in *C.* ×*hytivus*. This study helps to elucidate the meiotic properties and evolution of nascent allopolyploids with the dysploid parental karyotypes.

## Background

Interspecific hybridization and allopolyploidization frequently result in a ‘genomic shock’ that causes rapid genetic and epigenetic changes, due to the merging of two or more divergent and hybridizable genomes [1–3]. The meiosis abnormality of newly formed allopolyploids, as an immediate consequence of allopolyploidization, results in extensive abnormal chromosome pairing, imbalanced chromosome segregation and karyotype variations [4–6]. Cytogenetic studies have shown that different subgenomes have distinct meiotic behavior stabilities, and individual chromosomes have biases for chromosome loss and/or gain [7–9]. The extent of meiotic instability, genome structural and genetic/epigenetic changes may vary considerably in different polyploid species, suggesting that these changes depend on the origin and evolutionary differences between parental species [10]. To date, most studies on genomic variations and meiosis evolution have been conducted in nascent allopolyploids with analogous subgenomic karyotypes, such as same or close subgenomic chromosome numbers [11]. However, less attention has been devoted to synthetic allopolyploids with dysploid parental karyotypes.

Molecular cytogenetics is indispensable for studying the evolution of the polyploid genome, and it can intuitively visualize the dynamics of the genome. Most significantly, chromosome painting based on fluorescence in situ hybridization (FISH) has been verified as a powerful tool for identifying chromosomes and investigating chromosome rearrangements during evolution [12, 13]. Unfortunately, the issue concerning chromosome identification and tracking meiotic behavior based on chromosome painting has been less thoroughly explored due to complicated chromosome synteny and the absence of suitable probes, especially in interspecific hybrids and allopolyploids. New strategies for developing chromosome painting probes in plants have been inspired by the development of plant whole genome sequencing projects and technical advances in DNA synthesis. Single-copy sequence-based chromosome painting has been developed and applied to identify individual chromosomes, track chromosome pairing and detect rearrangements in cucumber, potato, maize and poplar [14–19]. This approach greatly facilitates the identification of homoeologous chromosomes, diagnosis of chromosome abnormalities, and tracking of pairing behavior in interspecific hybrids and allopolyploids.

**Fig. 1:**
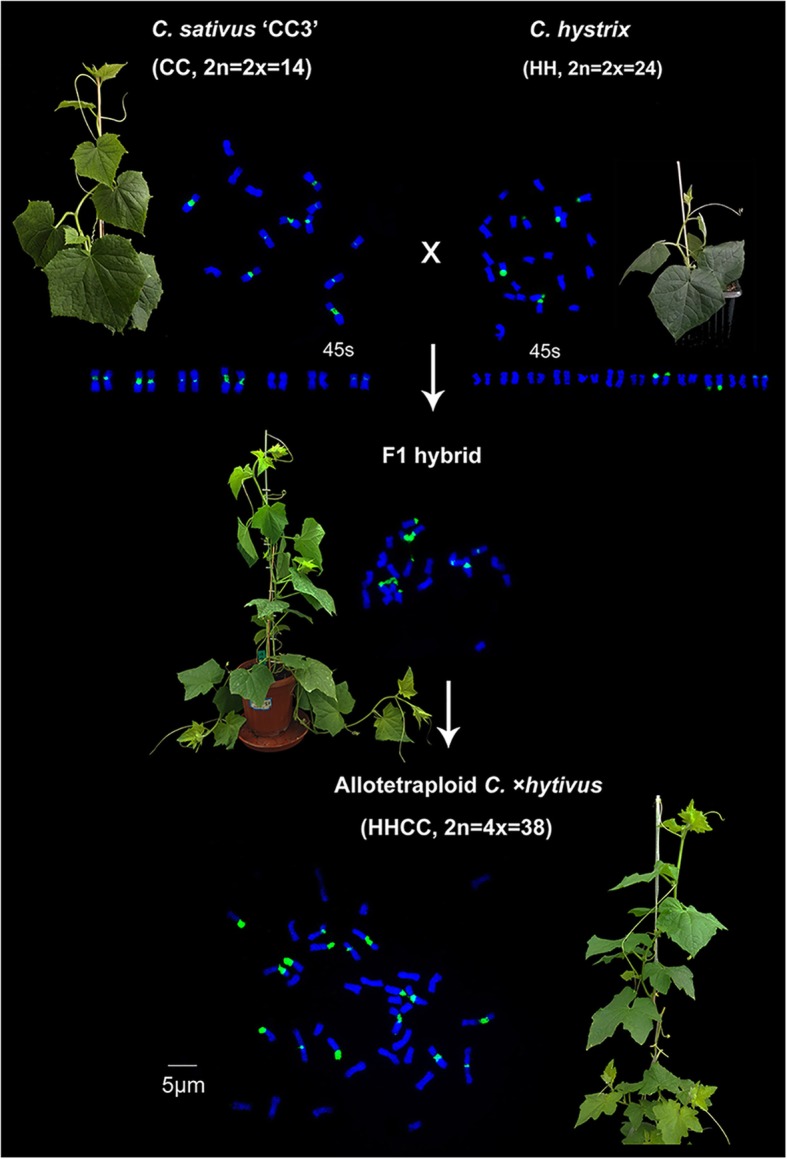
Synthesis strategy of allotetraploid *Cucumis ×hytivus*. The allotetraploid *Cucumis* ×*hytivus* (HHCC, 2n = 4x = 38) was synthesize through interspecific hybridization between cucumber (*Cucumis sativus* L. ‘BejingJietou’, CC, 2n = 2x = 14) and its sister species *C. hystrix* (HH, 2n = 2x = 24) followed by chromosome doubling. Mitotic metaphase chromosome patterns with 45S FISH signals were shown for each species

The allotetraploid *C.* ×*hytivus* J. F. Chen & J. H. Kirkbr. (HHCC, 2n = 4x = 38) was synthesized through interspecific hybridization between cucumber (*C. sativus* L. ‘BejingJietou’, CC, 2n = 2x = 14) and its sister species *C. hystrix* (HH, 2n = 2x = 24) followed by chromosome doubling [20, 21] (Fig. 1). Like other newly formed allopolyploids, the allotetraploid *C.* ×*hytivus* exhibits rapid genetic changes and extensive meiotic instability [11, 22, 23]. The cucumber and *C. hystrix* were derived from a common ancestral species via decreasing dysploidy (*n* = 12 to *n* = 7) approximately 4.6 million years ago [24, 25]. Different evolutionary fates have produced differences in karyotypes, genetics, cytological characteristics and specific traits between cucumber and *C. hystrix*. The interspecific hybrid and allotetraploid *C.* ×*hytivus* with distinctive subgenomic karyotypes provided a new means of revealing the genetic relationships between parental species compared to neo-allopolyploids with analogous subgenomic karyotypes, determining whether the synthetic allotetraploid *C.* ×*hytivus* exhibits novel meiotic properties and elucidating how to achieve stable meiosis.

We developed two oligo-probe pools from cucumber chromosomes 5 (C5) and 7 (C7) to identify homoeologous chromosomes, detect chromosomal rearrangements and track individual chromosome meiotic pairing in F_1_ hybrid and its derived allotetraploid. We analyzed the meiotic properties of the F_1_ hybrids and two different generations of *C.* ×*hytivus*. The meiotic behavior of individual chromosomes was significantly different in homoeologous pairing and univalent formation. The meiotic behavior of these chromosomes (C5, H9, H10 and C7, H1) in S_14_ generation was improved in the absence of extensive chromosome reshuffling located on the given chromosomes. GISH experiments were performed to investigate the possible differences of two subgenomic meiotic behaviors in the S_4_ and S_14_ inbred families. The meiotic behavior biases between two subgenomes were observed in S_4_ generation, especially univalent formation, chromosome lagging and asynchronous meiotic rhythm. After 10 generations of self-pollination, we document the increased meiotic stability in S_14_ generation, including synchronous meiosis, increased normal bivalents and reduced lagging chromosome. In addition, many autosyndetic pairings of H-chromosomes were observed in the F_1_ hybrids. Given the clear parental genetic background, the synthetic allotetraploid *C.* ×*hytivus* could serve as a uniquely traceable system to explore allopolyploidization and meiotic evolution of allopolyploids with distinctive parental karyotypes.

## Results

### Identification of individual chromosomes using oligo-painting

**Fig. 2:**
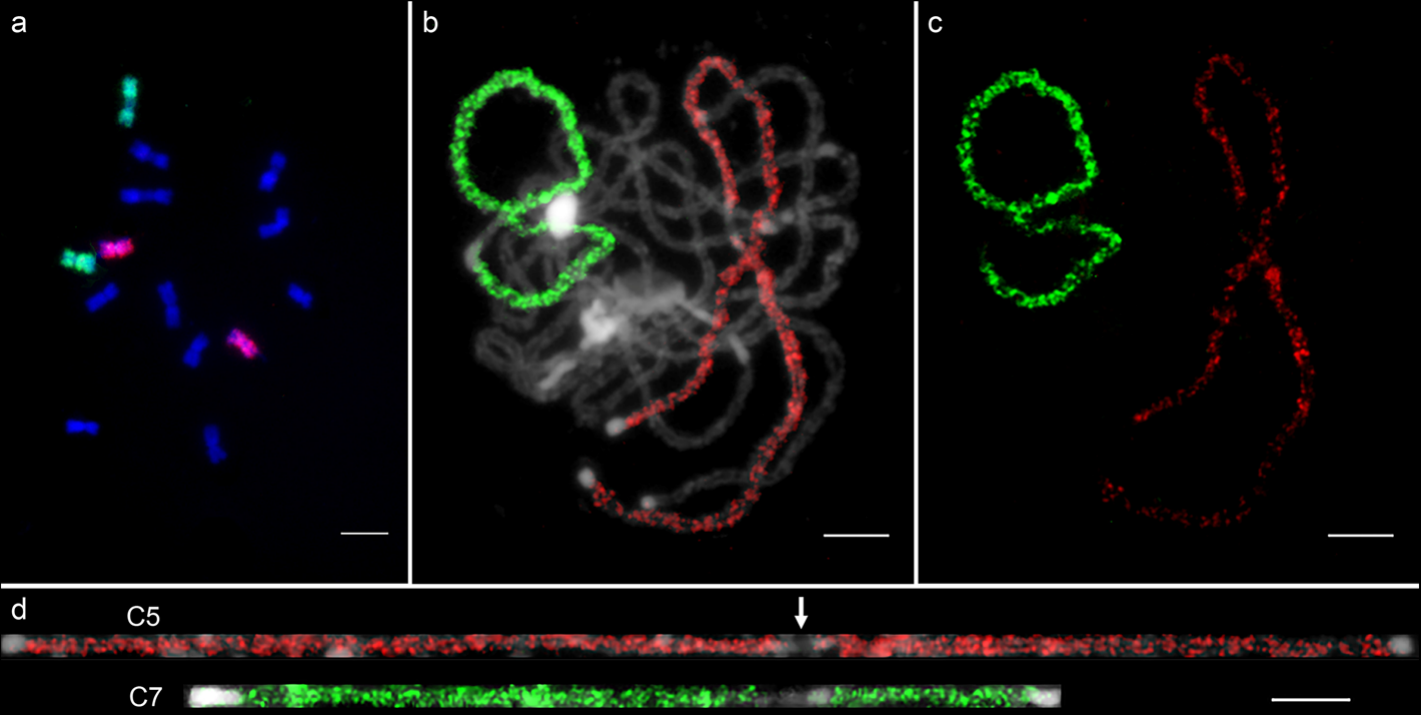
Oligo-based chromosome painting of chromosomes 5 and 7 in cucumber. **a** Painting of C5 (red) and C7 (green) on mitotic metaphase chromosomes. **b** Painting of C5 and C7 on meiotic pachytene chromosomes. **c** FISH signals were digitally separated from (**b**). **d** The straightened pachytene chromosomes C5 and C7 from (**b**). The white arrow indicates large signal gaps, including filtered centromeric region and 45 s rDNA locus. Bars = 5 μm

To study the meiotic behavior of individual chromosomes in interspecific hybrid and allotetraploid *C.* ×*hytivus*, we developed two oligo-painting probe pools of C5 and C7 based on the cucumber genome (see Materials and Methods). The average oligo densities of the two painting probes were 0.86 (C5) and 1.32 (C7) oligos per kilobase, respectively (Additional file 1: Figure S2). As expected, both probes produced bright and nearly uniform FISH signals on mitotic metaphase and meiotic pachytene chromosomes of cucumber (Fig. 2a, b). No extra oligo-FISH signals were detected on any other chromosomes (Fig. 2c), indicating that these two probes were highly specific to C5 and C7. There was an unambiguous and large signal gap on the pachytene chromosomes of C5 and C7 (Fig. 2d), which contain filtered centromeric regions and 45 s rDNA loci.

**Fig. 3:**
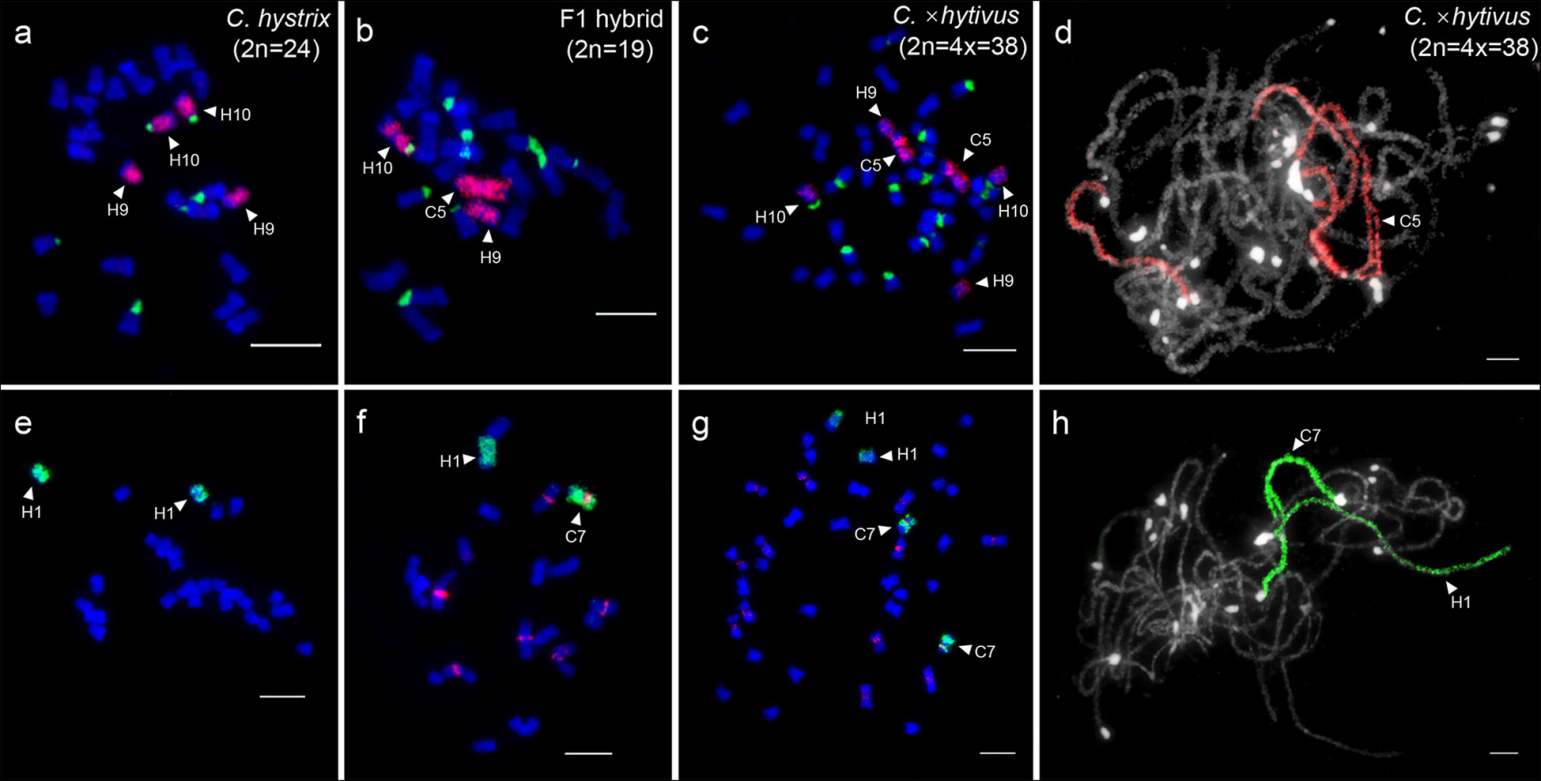
Identification of homoeologous chromosomes of C5 and C7 in *C. hystrix*, F_1_ hybrid and allotetraploid *C.* ×*hytivus*. **a**, **b**, **c** Painting homoeologous chromosomes H9 and H10 using C5-oligo probes (red) and 45 s rDNA probes (green) in *C. hystrix*, F_1_ hybrid and allotetraploid *C.* ×*hytivus*, respectively. **e**, **f**, **g** Painting homoeologous chromosome H1 using C7-oligo probes (green) and Type III probes (red) in *C. hystrix*, F_1_ hybrid and allotetraploid *C.* ×*hytivus*, respectively. **d** Individual chromosomes painting on meiotic pachytene using C5-oligo probes (red). **h** Individual chromosomes painting on meiotic pachytene using C7-oligo probes (green). Bars = 5 μm

The homoeologous chromosomes of C5 and C7 were identified by C5- and C7-oligo probes, Type III and 45 s rDNA probes at mitosis metaphase of *C. hystrix*, F_1_ hybrid and allotetraploid *C.* ×*hytivus* (Fig. 3). Oligo-FISH signals from C5-oligo probes were unambiguously detected on two *C. hystrix* chromosomes, 9 (H9) and 10 (H10), which were distinguished by oligo-FISH intensity and 45 s rDNA signal located on the pericentromeric region of H10 (Fig. 3a, b, c) [11, 25]. The *C. hystrix* chromosome 1 (H1) was painted by C7-oligo probes, which were distinguished by cucumber-specific Type III centromere probes (Fig. 3e, f, g). Then, the two oligo-probes were hybridized to meiotic pachytene chromosomes of S_14_ generation of *C.* ×*hytivus* (Fig. 3d, h). No unambiguous signal gaps were observed on meiotic pachytene chromosomes. Consequently, these chromosome painting patterns can rapid identify individual chromosomes of C5, C7, and H1, H9, H10, diagnose karyotypic variation and trace meiotic pairing of these chromosomes in F1 hybrids and allotetraploid *C.* ×*hytivus*.

### Homoeologous pairing and autosyndetic pairing in F_1_ hybrid

**Fig. 4:**
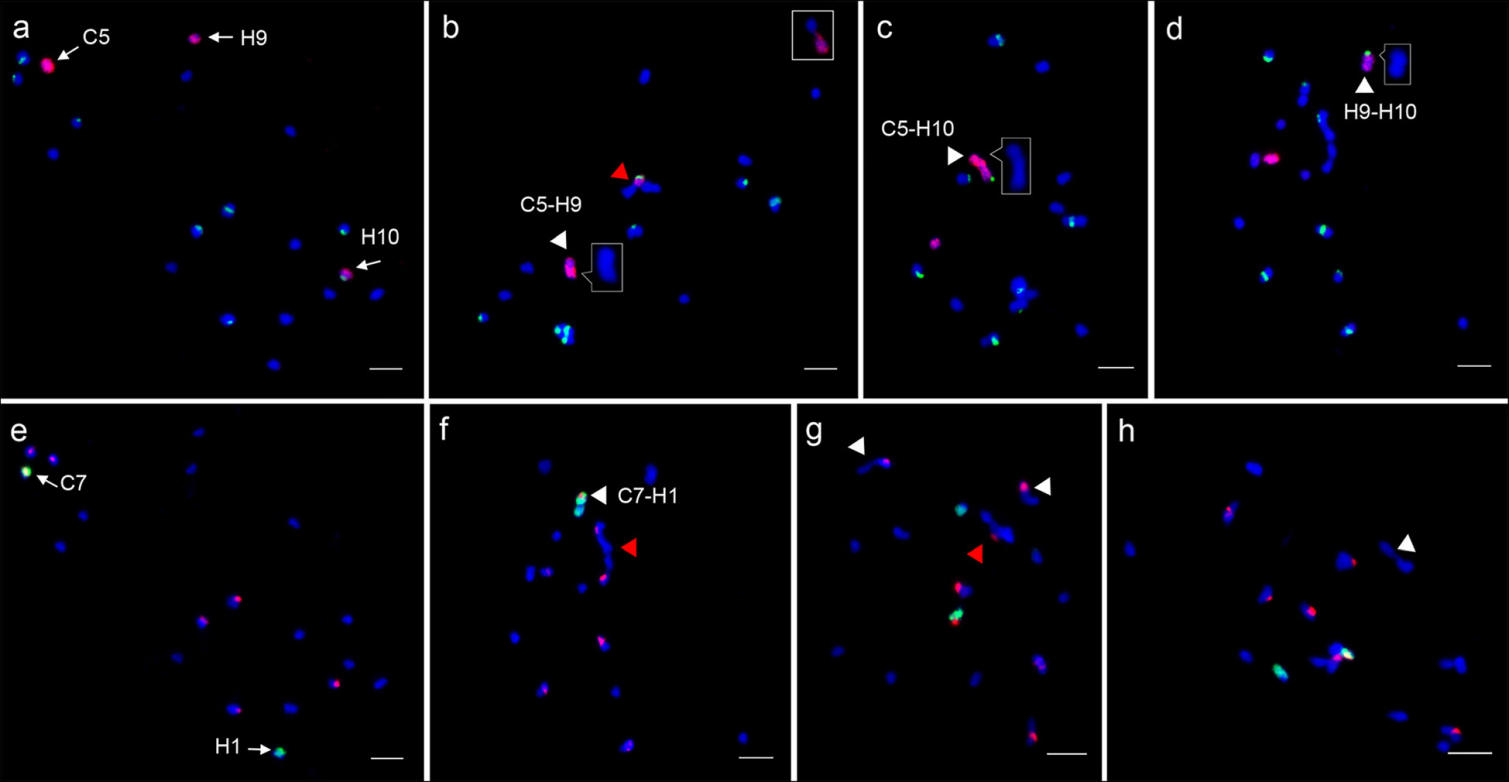
Tracing individual chromosome pairing in MI PMCs of the F_1_ hybrid. **a-d** The pairing behavior of chromosomes C5, H9 and H10 tracked by C5-oligos (red) and 45S rDNA probes (green). **a** The chromosomes C5, H9 and H10 as univalents. **b** Homoeologous pairing of C5 and H9 (white arrow). The pairing of H9 with another chromosome is shown in the box. H10 was paired with another chromosome (red arrow). **c** Homoeologous pairing of C5 and H10 (white arrow). **d** The chromosomes H9 and H10 were mispairing or connected together to form autosyndetic pairing (white arrow). All pairing configurations were enlarged in insets without FISH signals. **e-h** The pairing behavior of chromosomes C7 and H1 tracked by C7-oligo (green) and Type III probes (red). **e** The chromosomes C7 and H1 as univalents. **f** Homoeologous pairing of C7 and H1 (white arrow), a C-H-H-C intergenomic quadrivalent (red arrow). **g** Two intergenomic bivalents (white arrows) and one H-C-H trivalent (red arrow). **h** Two H-chromosomes formed an autosyndetic pairing (white arrow). Bars = 5 μm

Interspecific hybrids provide a new route for studying the genetic relationships among parental chromosomes through the assessment of meiotic chromosome pairing. We investigated the meiotic pairing of C5 and C7 and their homoeologous chromosomes after interspecific hybridization based on the oligo-FISH patterns described above (Fig. 4). We analyzed 132 and 106 well-resolved PMCs at meiotic metaphase I (MI) taken from four F_1_ plants to trace chromosome pairing using C5 and C7-oligo probes, respectively (Table 1). The C5, H9 and H10 were completely unpaired as univalents in 74 (56.1%) PMCs (Fig. 4a). We found that 30 (22.7%) PMCs harbored intersubgenomic bivalents of C5 and H9 (Fig. 4b), and 17 (12.9%) PMCs harbored bivalents of C5 and H10 (Fig. 4c). The C7 and H1 were completely unpaired as univalents in 87 (82.1%) PMCs (Fig. 4e). The bivalents of C7 and H1 were found in 19 (17.9%) PMCs (Fig. 4f, white arrow). Similarly, one to three intergenomic bivalents were detected in 45 (42.5%) PMCs (Fig. 4g, white arrows). The quadrivalent and trivalents were also detected in one and eight PMCs, respectively (Fig. 4f, g, red arrows).

**Table 1:** Meiotic chromosome behavior of five chromosomes at metaphase I and anaphase I in F_1_, S_4_ and S_14_ generations

Plant group	No. of PMCs at MI		No. (%) of PMCs with homologous bivalents		No. of PMCs with univalents		No. of PMCs with homoeologous pairings	
	C5-oligo	C7-oligo	C5/H9/H10	C7/H1	C5/H9/H10	C7/H1	C5 and H9/H10	C7 and H1
F1	132	106	–	–	86/92/103	87/87	29/17	19
S4	123	112	75 (61)	101 (90.2)	16/9/38	6/7	0/11	0
S14	137	104	119 (86.9)^**^	100 (96.2)^*^	5^**^/3^*^/14^***^	2/2^*^	0/3	0

*MI* metaphase I, *AI* anaphase I, *PMC* pollen mother cell; statistical test for comparisons with S4 (*t*-test): ^*^, *p* < 0.05; ^**^, *p* < 0.01; ^***^ *p* < 0.001

Interestingly, we found that 10 (7.6%) PMCs contained the autosyndetic pairings of H9 and H10 (Fig. 4d, inset). The pairing of H9 and another chromosome was detected in one PMC (Fig. 4b, box), whereas the pairing of H10 and another chromosome was found in two PMCs (Fig. 4b, red arrow). Two H-chromosomes (chromosomes from the H-genome) were autosyndetic pairing as a bivalent configuration in 27 (25.5%) PMCs (Fig. 4h). However, autosyndetic pairing of C-chromosomes was not observed in the 106 PMCs investigated. Considering each subgenome as a whole, the H-subgenome showed a higher autosyndetic pairing frequency. These results suggest that the meiotic behavior of the H-genome is more susceptible to the shock of interspecific hybridization than is the C-genome.

### Two translocations and possible cytological evidence detected based on oligo-painting

**Fig. 5:**
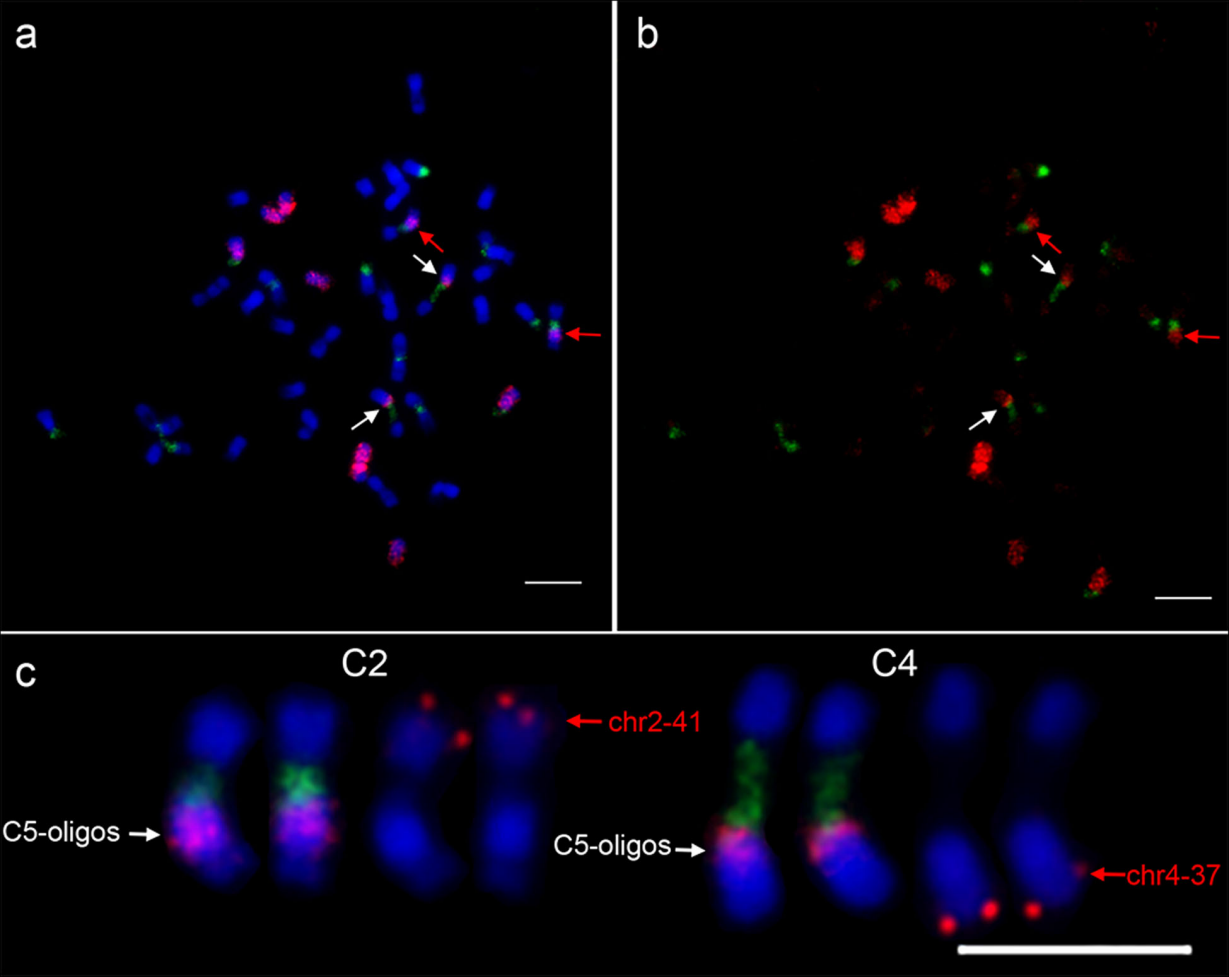
Two translocations carrying C5-oligo signals were detected based on oligo-painting in one S_14_ individual of *C.* ×*hytivus*. **a** Extra C5-oligo signals (red) were detected on two chromosomes carrying a large 45S rDNA probes (green) (white arrows). **b** FISH signals were digitally separated from (**a**). **c** Two chromosomes (C2 and C4) with extra C5-oligo signals were identified by two cucumber fosmid clones (chr2–41 and chr4–37). Bars = 5 μm

In our previous research, aneuploids or large-scale chromosomal rearrangements were not detected in 15 individuals from the S_13_ generation of allotetraploid *C.* ×*hytivus* based on the fosmid-FISH results [11]. This result might be attributed to few the individuals investigated, and the fosmid clones did not cover potential chromosomal rearrangement regions. We analyzed chromosomal variations via C5- and C7-oligo-painting in eight S_4_ and 36 S_14_ allotetraploid individuals. No visible chromosomal rearrangement events were observed in any individuals, except one S_14_ plant, in which there were two large translocations detected (Fig. 5, Additional file 1: Figure S3). Extra C5-oligo signals were detected on the long arm and near the centromeric region of two pairs of chromosomes with large 45 s rDNA locus (Fig. 5a, b). According to the karyotype of *C.* ×*hytivus* [11], we selected two fosmid clones (chr2–41 and chr4–37) to identify the two chromosomes with extra C5-oligo signals (Fig. 5c). We did not detect extra C7-oligo signals localized on other chromosomes, indicating that the chromosomes C7 and/or H1 do not experience nonhomologous rearrangements with other chromosomes in the analyzed plants.

**Fig. 6:**
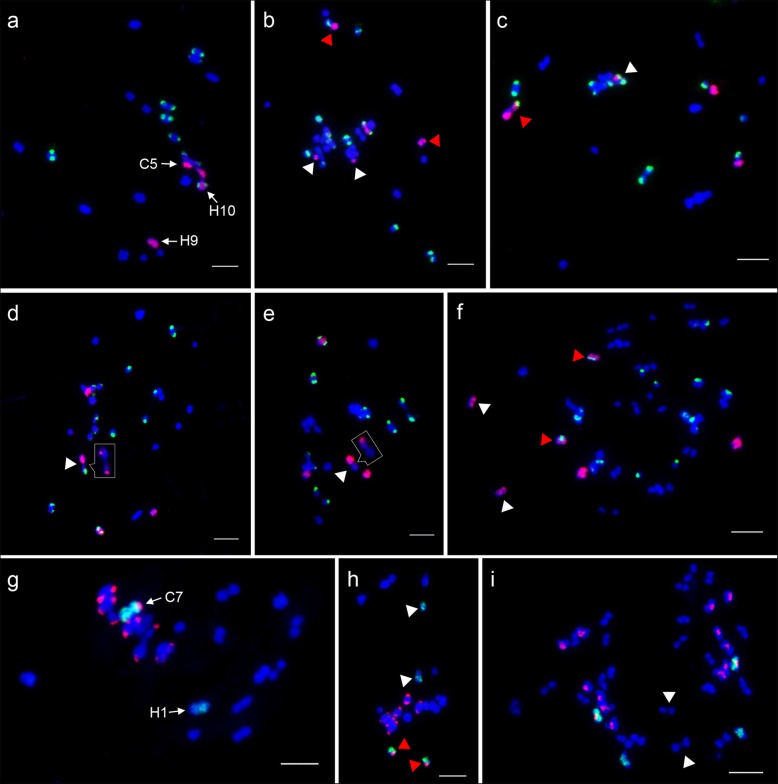
Tracking meiotic behavior of individual chromosomes at meiotic metaphase I and anaphase I in S4 generation. **a**-**f** The meiotic behavior of C5 H9 and H10 were traced based on C5-oligo probes (red) and 45 s rDNA probes (green). **a** Three exclusive homologous bivalents of C5, H9 and H10. **b** Two C5 univalents (red arrows) and two H9 univalents (white arrows). **c** A homoeologous pairing of C5 and H10 (red arrow) and one H10 univalent (white arrow). **d** The C5 formed an abnormal bivalent configuration with one C-chromosome with large 45 s rDNA. **e** The C5 formed an abnormal bivalent configuration with one H-chromosome. The identity of two chromosomes could be diagnosed by combining with Type III probes (red) (insets). **f** The H9 and H10 were unequal segregation at anaphase I. **g-i** The meiotic behavior of C7 and H1 were traced based on C7-oligo probes (green) and Type III probes (red). **g** Two exclusive homologous bivalents of C7 and H1. **h** Two C7 univalents (red arrows) and two H1 univalents (white arrows). **i** Two H-chromosomes lagging at anaphase I (white arrows). Bars = 5 μm

Notably, mispairing of C5 and other chromosome was observed in two PMCs from the S_4_ generation (Fig. 6d, e). We performed successive FISH experiments using Type III probes to identify the two chromosomes. The FISH results indicated that the chromosome with strong 45 s rDNA loci belonged to the C-subgenome (Fig. 6d, inset), and the other chromosome belonged to the H-subgenome (Fig. 6e, inset). These abnormal pairings may cause potential chromosomal rearrangements that could be stably inherited into the subsequent generations. Three strong 45 s rDNA loci were located on three C-subgenome chromosomes C1, C2 and C4 in allotetraploid *C. ×hytivus* [11]. One of these chromosomes likely formed a mispairing configuration with C5 and caused chromosomal translocations, which provided possible evidence for the two translocations in one S_14_ individual (Fig. 5).

### Meiotic behavior biases of individual chromosomes

To explore possible meiotic pairing differences among chromosomes at meiosis I of allotetraploid *C.* ×*hytivus*, we performed oligo-painting on meiotic chromosomes at meiotic pachytene, MI and anaphase I (AI). We analyzed chromosome pairing in a total of 76 PMCs at the meiotic pachytene stage from the S_4_ generation (Additional file 1: Figure S4). The C5 and H9/H10 partially paired in 24 (31.6%) PMCs (Additional file 1: Figure S4a, b, c). Completely unpaired C7 chromosome strings were detected only in three PMCs (Additional file 1: Figure S4d), and the long arm of C7 and H1 were unpaired in five (6.6%) PMCs (Additional file 1: Figure S4e, g) and six (8.7%) PMCs (Additional file 1: Figure S4f), respectively. However, the C7 and H1 partially paired or fully paired were not observed in all PMCs, although C7 and H1 are highly syntenic. In addition, we observed a considerable amount of unpaired chromosome fragments and single chromosome strings in the PMCs of S_4_ generation (Additional file 1: Figure S4, red arrows), which may result in potential chromosome rearrangement events and univalents.

We examined the paring behaviors of five chromosomes (C5, H9, H10 and C7, H1) at MI and AI of meiosis in eight S_4_ individuals (Fig. 6). A total of 123 and 112 well-resolved MI PMCs was analyzed to trace the meiotic paring behavior of C5, H9, H10 and C7, H1, respectively (Table 1). The results showed that 61% of PMCs harbored exclusive homologous bivalents of the C5, H9 and H10 (Fig. 6a), and 90.2% of PMCs contained exclusive homologous bivalents of C7 and H1 (Fig. 6g). Then, we calculated the frequency of univalents and homoeologous pairings of five chromosomes at MI (Table 1). We found that these chromosomes showed clear differences in their propensities to be in univalent state and homoeologous pairing (Fig. 8f and Table 1). Specifically, among the five chromosomes, H10 showed the highest frequency of univalent formation, whereas both C7 and H1 showed the lowest frequency of univalent formation. Homoeologous pairing of C5 and H10 was observed in 11 (8.94%) PMCs (Fig. 6c). However, the homoeologous pairing of C5/H9 and C7/H1 as bivalents or multivalents was not observed in all PMCs. Therefore, these chromosomes are biases for homoeologous pairing and univalent formation. Unequal segregation of H9 and H10 was detected at AI (Fig. 6f). Similarly, lagging H-chromosomes were also detected at AI (Fig. 6i).

### Meiotic chromosome biases of the subgenome cause the low fertility of allotetraploid *C. ×hytivus*

**Fig. 7:**
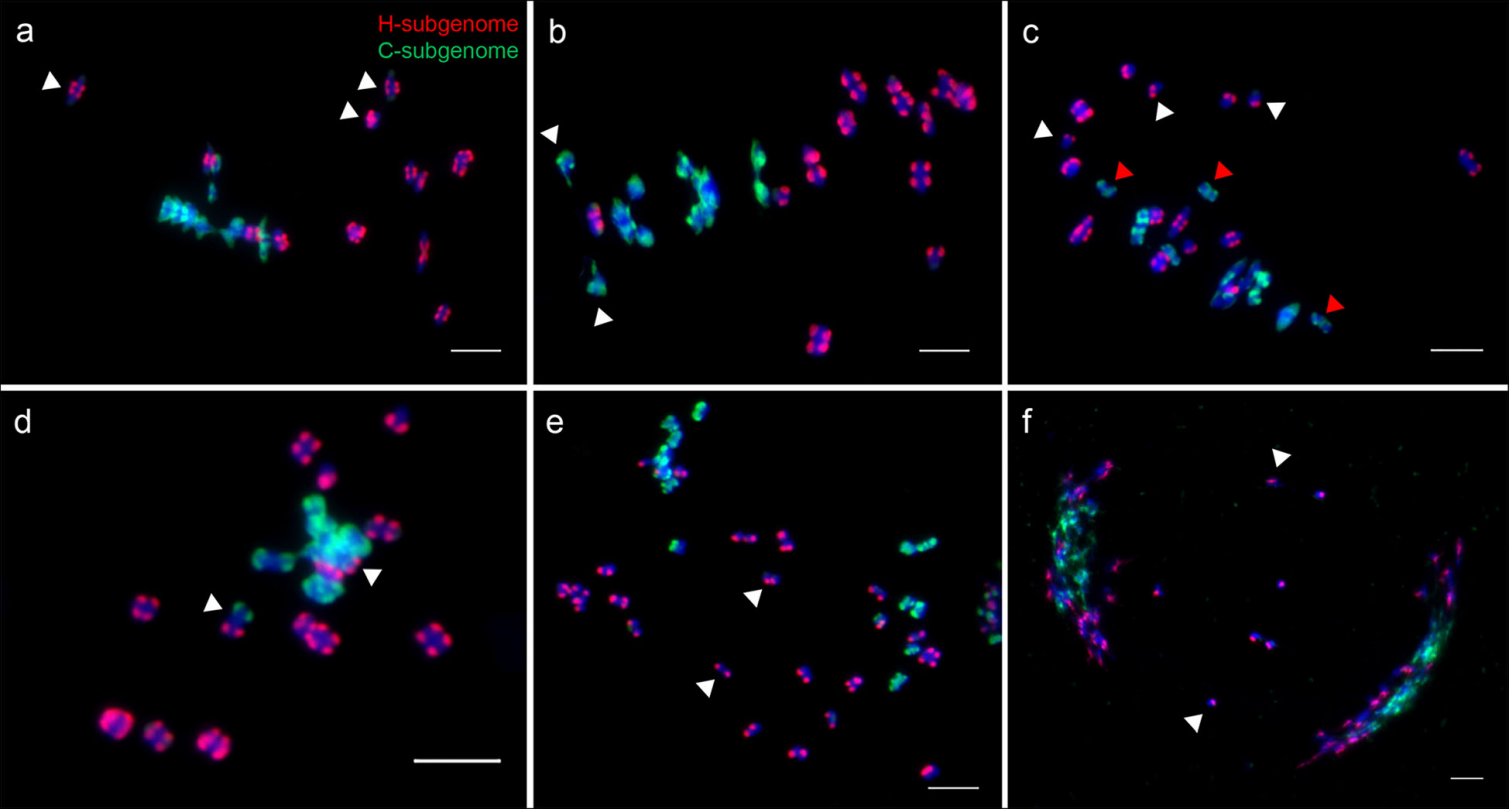
Representative abnormal meiotic behavior in S_4_ generation of allotetraploid *C. ×hytivus*. **a** Genomic in situ hybridization (GISH) image showing 19 homologous bivalents at metaphase I, H-subgenome (red) and C-subgenome (green). Six H-bivalents lagged behind C-bivalents (white arrows, partially indicated). **b** The disjunction of C-bivalents was earlier than that of H-bivalents (white arrow). **c** Six H-univalents (white arrows, partially indicated) and four C-univalents (yellow arrows, partially indicated). **d** Two intergenomic bivalents (white arrows). **e** and **f** Two examples of lagging H-chromosomes at anaphase I and telophase I (white arrows). Bars = 5 μm

**Fig. 8:**
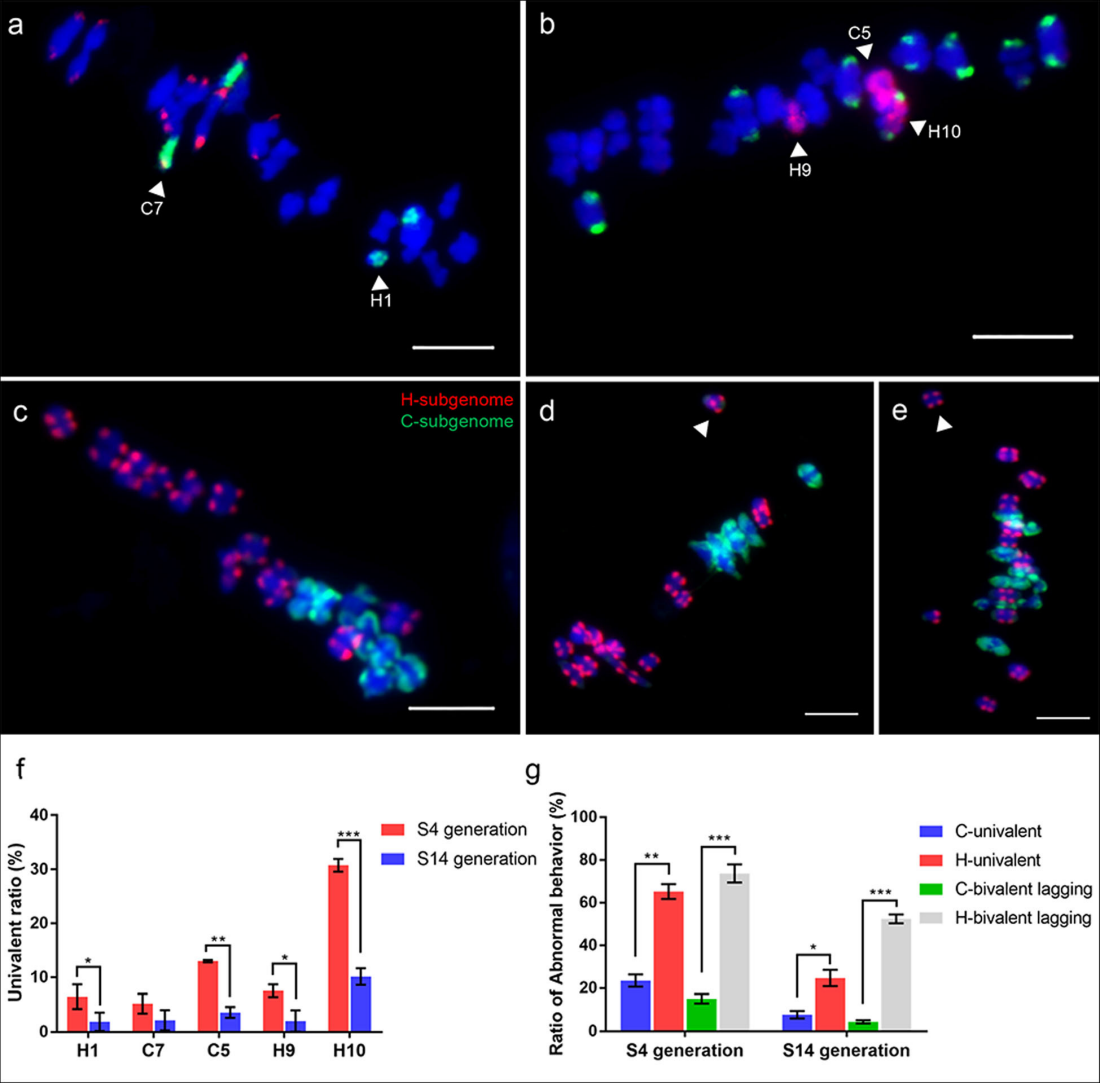
Meiotic behavior at metaphase I in S_14_ and the biases of chromosomes/subgenomes for univalents and lagged bivalents at metaphase I. **a** Two homologous bivalents of C7 and H1 at metaphase I in S_14_ generation. **b** Three homologous bivalents of C5, H9 and H10 at metaphase I in S_14_ generation. **c** GISH image showing normal meiotic behavior of two subgenomes at metaphase I in S_14_ generation. **d** and **e** Only one H-bivalent lagged behind the other bivalents and did not reach the equatorial plate at metaphase I (white arrows). Bars = 5 μm. Five chromosomes (H1, C7, C5, H9 and H10) and subgenome biases for the occurrence of univalents and lagging bivalents at MI of S_4_ and S_14_ were quantified by oligo-painting and GISH and are presented in (**f**) and (**g**) respectively. The x-axes in (**f**) and (**g**) refer to the five chromosomes and the different generations (S_4_ and S_14_) respectively, while the y-axes in (**f**) and (**g**) refer to the frequency of univalent(s) and lagged bivalent(s). Error bars indicate the ±SD over three biological replicates. Asterisks indicate statistically significant differences at *, *p* < 0.05; **, *p* < 0.01; *** *p* < 0.001

We investigated possible differences in the meiotic behavior between two subgenomes in MI and AI PMCs taken from eight S_4_ and ten S_14_ generations through GISH experiments (Table 2). We observed many PMCs of S_4_ generation with clear abnormalities, including the presence of asynchronous meiosis at MI (Fig. 7a, b), univalents (Fig. 7c), intergenomic pairings (Fig. 7d, Additional file 1: Figure S5) and lagging chromosomes at AI/TI (Fig. 7e, f). As expected, most of the chromosomes still maintained bivalent configurations in each PMC. Notably, most C-bivalents could be precisely and tightly aligned on the equatorial plate, whereas the H-bivalents seemed irregularly dispersed or lagged behind the C-bivalents at MI (Fig. 6g, Fig. 7a). Some of the H-bivalents were just arrived at the equatorial plate when the C-bivalents had already begun to segregate (Fig. 7b). Furthermore, 73.4% of the MI PMCs showed lagged H-bivalents, which was significantly higher than the 15.4% of PMCs with lagged C-bivalents in S_4_ generation (Fig. 8g). Similarly, the frequency of lagged H-bivalents was also significantly higher than that of C-bivalents in the S_14_ generation (Fig. 8g). These results indicated that the meiosis of two subgenomes was significantly asynchronous; that is, the H-subgenome required more time to complete the meiosis process than the C-subgenome in one nucleus.

**Table 2:** Meiotic chromosome behavior in S_4_ and S_14_ generations of the synthetic allotetraploid *Cucumis ×hytivus*

Plant group	No. of PMCs at MI	No. (%) of PMCs with 19 homologous bivalents	No. (%) of PMCs with univalents		No. (%) of PMCs with lagged bivalents		No. (%) of PMCs with inter-genomic pairings	No. of PMCs at AI	No. (%) of PMCs with chromosome lagging	
			C	H	C	H			C	H
S_4_	143	49 (34.3)	34 (23.8)	93 (65.0)	22 (15.4)	105 (73.4)	35 (24.5)	95	26 (27.4)	75 (79.0)
S_14_	131	98 (74.8)^**^	9 (6.9)^**^	31 (23.7)^*^	6 (4.6)^**^	68 (51.9)^**^	13 (9.9)^**^	113	11 (9.7)^*^	63 (55.8)^*^

*MI* metaphase I, *AI* anaphase I, *PMC* pollen mother cell, *C* C-subgenome, *H* H-subgenome; statistical test for comparisons with S_4_ (*t*-test): ^*^, *p* < 0.05; ^**^, *p* < 0.01

The H-subgenome showed significantly higher univalent formation and lagging frequencies than the C-subgenome in both the S_4_ and S_14_ generations (Table 2). The results indicated that the propensities of the two subgenomes for meiotic behavior were significantly different. The meiotic behavior of the H-subgenome is more responsive to allopolyploidization, which eventually contributed to extensive H-chromosome lagging at subsequent stages (Table 2, Fig. 7e, f). Lagged chromosomes caused male gametes with incomplete chromosome complements, which resulted in the low pollen fertility of allotetraploid *C. ×hytivus*. In conclusion, meiotic behavior biases of two subgenomes are the primary factor for the low fertility of allotetraploid *C. ×hytivus*.

### Increased meiotic stability by harmony between two subgenomes

We examined the meiotic behavior of C5, H9, H10 and C7, H1 in 137 and 104 well-resolved PMCs, respectively, taken from ten S_14_ plants with no nonhomologous chromosomal rearrangement based on C5/C7 oligo-painting (Table 1). Compared with the S_4_ generation, the frequency of homologous bivalents of the five chromosomes was significantly improved in the S_14_ generation (Table 1, Fig. 8a, b). Correspondingly, the univalent frequencies of H1, C5, H9 and H10 were significantly lower than those of S_4_ generation (Fig. 8f). The chromosome H10 still had the highest univalent frequency among the five chromosomes (Fig. 8f). The univalent frequencies of C7 between the S_4_ and S_14_ generations were statistically insignificant (Fig. 8f). These results indicated that the meiotic stability of these chromosomes was improved in the absence of extensive chromosome reshuffling located on the given chromosomes.

In analyzed 131 well-resolved MI PMCs from the S_14_ generation, 98 (74.8%) of PMCs contained 19 exclusive homologous bivalents, which was significantly higher than 49 (34.3%) detected in the S_4_ generation (Table 2). The frequencies of meiotic abnormalities in the S_14_ generation were remarkably reduced compared with the S_4_ generation (Table 2, Fig. 8c), including univalents, intergenomic pairing and chromosome lagging. Importantly, the frequency of MI PMCs containing asynchronous meiosis was significantly reduced in the S_14_ generation (Table 2). In MI PMCs with asynchronous meiosis, only a few (one to three) H-bivalents have not yet reached the equatorial plate in S_14_ generation (Fig. 8d, e), which was significantly reduced compared with S_4_ generation with one-seven lagged H-bivalents (Additional file 1: Figure S6). The results showed that two subgenomes have achieved a certain degree of synchronous meiosis at MI. Predictably, the pollen fertility of S_14_ plants was prominently higher compared to S_4_ plants, but still remained at a relatively low level (less than 50% on mean) (Additional file 1: Figure S1). These results demonstrated that meiosis stability can be increased through continuous selfing, and harmony between subgenomes.

## Discussion

### Meiotic affinities of homoeologous chromosomes

Evaluating homoeologous pairing in interspecific hybrids can provide important information for the affinities among homoeologous chromosomes [26]. The pairing preferences between chromosomes and their potential partners pay less attention in interspecific hybrids and allopolyploids due to the absence of FISH technology to identify individual chromosomes. The cucumber chromosome C5 originated from fusion of inferred chromosomes AK9 and AK10 (ancestor karyotype) similar to H9 and H10 of *C. hystrix*. Cucumber chromosome C7 and *C. hystrix* chromosome H1 are evolved from the ancestor chromosome AK1. These chromosomes had experienced strikingly different evolutionary fates [25, 27]. In the analyzed PMCs of F_1_ hybrids, the homoeologous pairing of C5 and H9 (22.7%) had a higher frequency than pairing of C5 and H10 (12.9%), which may be explained by the collinearity structure of C5 subtelomeric regions with H9 [25]. When an individual chromosome has multiple homoeologous chromosomes, these chromosomes may be sorted by preferential chromosome pairing, because the recognition among chromosomes occurs preferentially between subtelomeric regions [28, 29]. The homoeologous pairing of C7 and H1 was observed only in 19 (17.9%) MI PMCs of the F_1_ hybrid, even though C7 is highly conserved with H1 of *C. hystrix*. Therefore, the pairing affinities among homoeologous chromosomes may be attributable to accumulated changes in the chromosomal structure during evolution.

Interestingly, 7.6% of PMCs containing autosyndetic pairing of H9 and H10 were observed in the F_1_ hybrid. H9 and H10 paired with other H-chromosomes only in one and two PMCs, respectively. Pairing of H1 and other H-chromosomes were not observed in all PMCs. The autosyndetic pairings within one genome implied that segmental homology and/or common origin between the involved chromosomes [30]. The results indicated that H9 and H10 have a certain meiotic affinity and that H1 has a lower meiotic affinity with other H-chromosomes. Meiotic behavior of these chromosomes is dramatically different in response to interspecific hybridization, which appears to be related to the evolutionary fates of these chromosomes. The AK9 and AK10 have two distinct evolutionary fates: evolving into one chromosome (C5) after the fusion event or forming present the H9 and H10 [25]. The meiotic affinity between H9 and H10 may increase our understanding of the fusion event of AK9 and AK10.

Among the five chromosomes analyzed in two different generations of allotetraploid *C. ×hytivus*, H10 showed a higher univalent frequency than other chromosomes. H10 with an asymmetric centromere position (acrocentric) may form an unstable bivalent, because a certain arm length is required for stable bivalent formation [31]. Unpaired H10 may disturb the pairing of C5, causing C5 to have a relatively high univalent frequency and pairing with H10. Our results showed that the univalent frequencies of C7, H1 and H9 are relatively lower with no C7-H1 and C5-H9 pairings. These results suggest that C7, H1 and H9 can normally recognize homologous chromosomes and form stable bivalents. Homoeologous pairing and recombination are attributed to not only the chromosome structure but also the interaction of genetic factors, such as *ph 1* in allohexaploid wheat, *PrBn* in allotetraploid *Brassica napus*, and *BYS* in allotetraploid *Arabidopsis suecica* [5]. However, the pairing control locus may be insufficient to ensure the exclusive diploid-like meiotic chromosome pairing of newly formed allopolyploids, and additional modifications are still needed in meiotic stabilization [9]. In addition, the timing of chromosome condensation also affects chromosome pairing [32].

### Subgenome biases in meiotic chromosome behavior

Interspecific hybrids and allopolyploids face many challenges caused by large-scale conflict among divergent parental genomes, commonly referred to as ‘genomic shock’ associated with a de novo merger of two or more divergent parental genomes. Because unique evolutionary fates shaped each parent, producing species-specific karyotypes, genetic and epigenetic differences [1]. In interspecific hybrids and allopolyploids, subgenome dominance appears to be a common result that responds differentially to genomic shock, such as subgenome biases in epigenetic regulation, gene expression and homoeologous exchanges [1]. Similarly, the meiotic chromosome behavior of subgenomes also has biases; for example, a subgenome exhibits higher univalent and chromosome lost frequencies or maintains relatively stable meiotic behavior.

The autosyndetic pairing, which is involved in chromosomes from the same genome, was observed in several *Paphiopedilum* and *Brassica* interspecific hybrids [33, 34]. Similarly, many PMCs (25.5% of 106 PMCs) containing H-chromosome autosyndetic pairings were observed in the F_1_ hybrid. However, autosyndetic pairing was not observed between any two C-chromosomes. These results suggest that the meiotic pairing of two genomes has biases in the F_1_ hybrid. The cucumber and *C. hystrix* have derived from an ancestral species via the mechanisms of decreasing dysploidy (*n* = 12 to *n* = 7), leading to a high level of evolutionary differences between two genomes [24, 25]. The magnitude of genomic changes in response to genomic shock appears to be correlated with the degree of subgenome evolutionary differences [1]. The autosyndetic pairing of H-chromosomes indicates that the meiotic behavior of the H-genome may be more susceptible by interspecific hybridization than the C-genome in the F_1_ hybrid. Therefore, the different responses of the H- and C-genomes to genomic shock or interspecific hybridization may be attributed to the evolutionary differences between the two parental species.

Extensive cytological studies have shown that homologous bivalents are the dominant pairing configuration in the meiosis of allopolyploids, and individual chromosome/subgenomes have biases in terms of meiotic behavior in some allopolyploids [5, 7, 9]. The meiotic behavior biases among subgenomes were observed in synthesized allohexaploid wheat and *Brassica*. The distinct subgenome stability was B > A > C in *Brassica* allohexaploid and D > A > B in synthesized wheat allohexaploid; that is, the C-subgenome of *Brassica* allohexaploid and the B-subgenome of wheat allohexaploid showed the highest frequencies of univalent formation and chromosome loss [7–9]. Indeed, the meiotic behavior biases between two subgenomes were also observed in allotetraploid *C. ×hytivus*, including asynchronous meiosis, univalent formation, and lagging chromosomes. The meiotic stability is C-subgenome > H-subgenome in *C. ×hytivus*, the H-subgenome showed higher univalents and chromosome lagging frequencies. However, no obvious asynchronous rhythm was observed in some newly formed allopolyploids, such as synthetic wheat allotetraploid and allohexaploid [9, 35], *Brassica* allotetraploid and allohexaploid [7, 34]. The asynchronous meiotic rhythm between subgenomes, originating from differences in progression through the meiotic cell cycle between parental subgenomes [36, 37], may be attributed to distinctive karyotypes and evolutionary differences of parental species. The H-subgenome requires a long duration of the cell cycle to complete meiosis, and is a major cause of the high frequency of H-chromosome lagging in *Cucumis* allotetraploid [38]. Only 9.9% of MI PMCs and 9.7% of AI PMCs contained intergenomic pairing and C-chromosome lagging in S_14_ generation, respectively. Conversely, 55.8% of AI PMCs contained H-chromosome lagging in S_14_ generation, indicating that the relatively unstable meiotic behavior of the H-subgenome is primarily responsible for the low fertility of allotetraploid *C. ×hytivus*. The meiotic behavior of the two subgenomes is dramatically different both in the F_1_ hybrid and *C. ×hytivus*, likely associated with the differences of karyotypes, inherent cytological characteristics and evolutionary history between two parental species.

### Meiotic harmony of two subgenomes is a key subject of meiotic stability in synthetic Cucumis allotetraploid

Extensive chromosome reshuffling and aneuploidy were observed in wheat allopolyploids [8, 35], *Brassica* allopolyploids [39] and *Tragopogon* neo-allopolyploids [40, 41]. However, no aneuploidy was detected in the analyzed allotetraploid *C. ×hytivus*, possibly because that the zygotes containing aneuploidy cannot develop into full shape seeds, and then were neglected when collecting seeds or selecting for germination. Notably, two large translocations and possible evidence were detected using oligo-based chromosome painting. However, the rearrangement events of C7/H1 and other chromosomes were not detected in this study. Our results imply that the potentials of individual chromosomes for chromosomal rearrangements and/or structural variations may be different during the process of polyploid evolution. Recently, a chimeric gene, responsible for delayed leaf maturation in the allotetraploid *C. ×hytivus*, was cloned, which originated from homoeologous recombination [42]. This result implies that the *Cucumis* allotetraploid experienced homoeologous exchanges or recombination. We also observed that many PMCs contained intergenomic pairing, which was the cytological basis of homoeologous exchanges in *Cucumis* allotetraploid. It is possible that the rearranged fragments and homoeologous exchanges containing beneficial genes from wild relatives, which can be traced by oligo-FISH, were transferred into crop cultivars through introgression breeding.

Given the subgenome biases for meiotic chromosome behavior, how to coordinate the meiotic behavior between two subgenomes has become a key subject of meiotic stabilization in allotetraploid *C. ×hytivus*. We analyzed the behavior of five chromosomes (C5, H9, H10 and C7, H1) in two generations, and found that the abnormal meiotic behavior of these chromosomes was improved in the absence of these chromosome reshuffling. Meiotic stability is significantly improved through continuous selfing process, including reduced univalent formation, intergenomic pairing and chromosome lagging. The most obvious evidence is that the two subgenomes achieved a certain degree of synchronization at MI of meiosis in the S_14_ generation (Additional file 1: Figure S6). The number of H-bivalents that did not reach the equatorial plate was significantly reduced in S14 generation compared with S_4_ generation. The meiotic stabilization mechanisms possibly accelerated in several early generations of *C. ×hytivus*, but still require many generations to stabilize. Similar to the situation in other neo-allopolyploid plant taxa, the synthesized allotetraploid *C. ×hytivus* also experienced rapid genetic and epigenetic changes [22, 23, 43, 44]. These changes laid the foundation for meiotic harmony between two subgenomes. The results of this study will provide an interesting case to explore the meiotic evolution in allopolyploids with distinctive subgenomic karyotypes.

## Conclusion

Taken together, the results of this study indicate that oligo-FISH is a powerful and efficient technique for chromosome identification, chromosome variation diagnosis, and tracing of meiotic behavior, especially in polyploids with complex genomes. In interspecific F_1_ hybrids and allotetraploids, the meiotic biases of five chromosomes (C5, H9, H10 and C7, H1) for homoeologous pairing and univalent formation may be attributed to these chromosomes pairing affinities and structure. The difference in the meiotic properties of the two genomes for autosyndetic pairing, asynchronous meiosis, univalent formation and chromosome lagging indicates that the two genomes differentially respond to interspecific hybridization and allopolyploidization. These differential responses are associated with differences in karyotypes, inherent cytological characteristics and evolutionary history between two parental species. Thus, the harmony of difference meiotic behavior between two subgenomes has become a key subject of meiotic stabilization in allotetraploid *C. ×hytivus*. Indeed, the meiotic stability increased in S_14_ generation, including synchronous meiosis, reduced univalent formation and chromosome lagging. Our analysis provided new insights into the meiotic properties and meiosis stabilization of nascent allotetraploids with dysploid parental karyotypes.

## Methods

### Plant materials

The plant materials used for this study included two diploid parents (*C. sativus* ‘CC3’ and *C. hystrix*), their interspecific F_1_ hybrid and synthetic allotetraploid C. ×*hytivus* (Fig. 1) [20, 21], provided by the state key lab of Cucurbit Genetics and Germplasm Enhancement of Nanjing Agricultural College. All different generations of C. ×*hytivus* were obtained from the same 3th (S_3_) inbred family, which was obtained from a single S_0_ plant of C. ×*hytivus* by selfing. The seeds (> 20) were randomly selected from each generation seed set for breeding the next generation by selfing. We currently obtain 14th generation through continuous selfing. Four different generations (eight individuals of 4th (S_4_), five individuals of 8th (S_8_), six individuals of 11th (S_11_) and 36 individuals of 14th (S_14_)) of C. ×*hytivus* were randomly chosen from last generation seed sets for pollen viability, karyotyping and meiosis analysis. The pollen viability analysis showed that the pollen fertility of S_14_ generation was significantly higher than that of S_4_ generation, and had a statistically highly significant difference (*P* = 0.0009). Therefore, we selected all S_4_ and S_14_ plants to conduct mitotic analysis based on C5 and C7-oligo probes. Four F_1_, eight S_4_ and ten S_14_ (randomly selected from 36 S_14_ plants) individuals were used for meiotic analysis based on oligo-FISH and GISH (Additional file 2 Table S1). All the materials were grown in a greenhouse at Baima Teaching and Research Base Modern Agricultural Science and Technology Zone of Nanjing Agricultural University, Nanjing, China.

### Chromosome preparation

Root tips and young male flower buds of all materials were collected and fixed in Carnoy’s solution at 4 °C for at least 1 day. Specifically, the root tips of 8 S_4_ and 36 S_14_ individuals were separately collected to detect chromosomal variations via chromosome painting. The young male flower buds of four F_1_ individuals were collected and mixed together multiple times during flowering. The eight S_4_ individuals and ten S_14_ individuals were divided into three groups. The young male flower buds of each group were respectively collected multiple times. One anther from each flower bud was dissected to examine the developmental stage of PMCs. The remaining anthers from the same flower bud were collected into the corresponding groups for chromosome preparation. The procedure of chromosome preparations was performed as described previously [11, 18] with some modifications. The fixed root tips were digested with an enzyme mixture containing 4% cellulose R-10 (Yakult), 2% pectinase (Sigma-Aldrich) and 0.1% pectolase (Yakult) in 0.01 M citrate buffer (pH = 4.8), at 37 °C for 40–60 min. The anthers were collected and digested using enzyme mixtures, including 4% cellulose R-10 (Yakult), 4% pectinase (Sigma-Aldrich) and 2% pectolase (Yakult) at 37 °C for 50–70 min (meiotic pachytene) and 2–3 h (meiotic metaphase and anaphase). Finally, these digested root tips and anthers were smeared onto slides as described previously [18]. The slides with well-spread chromosomes will be prepared for FISH and GISH experiments.

The cytoplasm was removed by pepsin treatment to facilitate penetration of the probes. The slides with well-spread chromosomes were treated as described protocol [45] with some modifications: washing slides two times in 2× SSC in a Coplin jar for 3 min, followed by treating with 0.1 mg/mL pepsin (Sigma-Aldrich) in 10 mM HCl at 37 °C for 40 s - 1 min, and washing two times in 2× SSC at room temperature for 3 min. Finally, postfix slides with 4% formaldehyde in 2× SSC for 10 min, followed by washing two times in 2× SSC for 5 min, dehydrating in 70, 90 and 100% ethanol for 5 min each, and being left to air-dry.

### Probe preparation and oligo-FISH

Previous studies indicated that C7 is highly conserved and preserves a complete synteny with *C. hystrix* chromosome H1, whereas C5 corresponds to two *C. hystrix* chromosomes H9 and H10 [11, 25]. The oligo-probes of C5 and C7 were developed using the oligo selection software Chorus (https://github.com/forrestzhang/Chorus) [14]. Briefly, the repetitive sequences in the cucumber ‘Chinese Long’ genome (ftp://cucurbitgenomics.org/pub/cucurbit/genome/cucumber/Chinese_long/v2/, v2 Genome) were filtered using RepeatMasker (http://www.repeatmasker.org/). The filtered C5 and C7 sequences were divided into oligos (45 nt) with a step size of 5 nt. Each oligo was aligned to the cucumber genome to filter out those with duplicates in the genome (> 75% similarity over all 45 nt). Oligo with dTm > 10 (dTm = Tm – hairpin Tm) were kept to build an oligo probe database. We adjusted the number of oligos across the chromosomes to ensure that the oligo probes can cover the entire chromosomes. Specifically, oligos targeting 100 kb per 300 kb were chosen for chromosomes C5 and C7 (Additional file 3: Table S2). A total of 27,392 oligos per oligo pool were synthesized de novo in parallel by Mycroarray (Ann Arbor, MI), and were labeled following published protocols [14, 19].

The two oligo probes and two satellite DNA sequences, Type III and 45 s rDNA, were used for identifying chromosomes at mitosis and meiosis. Genomic DNA was extracted from cucumber and *C. hystrix* using the CTAB method [46], which were then labeled as GISH probes for distinguishing two subgenomes at meiosis of allotetraploid C. ×*hytivus* [11]. All the experimental procedures for FISH were performed as previously described [14, 18]. The final images contrast was processed using ADOBE PHOTOSHOP CC (Adobe, http://www.adobe.com). The pachytene chromosomes in Fig. 2 were straightened using ImageJ software (https://imagej.nih.gov/ij/).

### Pollen viability

Five biological replicates were prepared for pollen viability. Fifteen male flowers were randomly collected from each generation of allotetraploid C. ×*hytivus* for each biological replicate. Pollen grains were collected and stained with modified Carbol-fuchsin solution, and more than 2000 pollen grains per biological replicate were counted under a stereomicroscope. The percentage of plump pollen grains was calculated to represent the pollen viability of allotetraploid C. ×*hytivus*.

### Statistical analysis

Meiotic behaviors of five chromosomes and two subgenomes were counted in Additional file 2: Table S1. Statistical tests for each comparison and graphical analysis were executed in GraphPad Prism 7 (https://www.graphpad.com). An F-test was used to test for differences in the ranges of SD, and the pairwise Student’s *t*-test was used for comparisons pollen viability of four different generations and meiotic chromosome behavior of two different generations.

## Supplementary information

**Additional file :1 Figure S1.** Comparison of pollen viability of four generations of C. ×*hytivus* allotetraploid (S_4_, S_8_, S_11_ and S_14_). **Figure S2.** Locations and density of 27,392 oligos along the sequence map of cucumber chromosomes 5 (a) and 7 (b). **Figure S3.** Two translocations carrying C5-oligo signals were detected in other PMCs from the same individual as Fig. 5. **Figure S4.** Tracing chromosome pairing at meiotic pachytene of S_4_ generation. **Figure S5.** Three representative PMCs with multivalents and one lagged C-bivalent at metaphase I in S_4_ generation. **Figure S6.** A heatmap depicting the frequency of different lagged bivalent numbers in the asynchronous meiotic PMCs of the S_4_ and S_14_ generation.

**Additional file 2: Table S1.** Meiotic analysis of F_1_, S_4_ and S_14_ plants based on oligo-painting and GISH.

**Additional file 3: Table S2.** Location and sequence information of C5- and C7-oligos.

## Data Availability

All relevant supporting data can be found within the Additional files accompanying this article. Additional file 3: Table S2 contains all information about the number and locations of selected oligos of cucumber chromosomes 5 and 7. The Chorus software used for oligo-FISH probe design is freely available (https://github.com/forrestzhang/Chorus).

## Abbreviations

AI: Anaphase I of meiosis
FISH: Fluorescence in situ hybridization
GISH: Genomic in situ hybridization
MI: Metaphase I of meiosis
Oligo: Oligonucleotides
PMC: Pollen mother cell
