# Supplementary material for: Oligo-painting and GISH reveal meiotic chromosome biases and increased meiotic stability in synthetic allotetraploid Cucumis ×hytivus with dysploid parental karyotypes
Source: BMC Plant Biol. 2019 Nov 6;19:471. doi: 10.1186/s12870-019-2060-z (PMC6833230; doi:10.1186/s12870-019-2060-z)
Supplement: Supplementary file 1 — Additional file :1 Figure S1. Comparison of pollen viability of four generations of C. ×hytivus allotetraploid (S4, S8, S11 and S14). Figure S2. Locations and density of 27,392 oligos along the sequence map of cucumber chromosomes 5 (a) and 7 (b). Figure S3. Two translocations carrying C5-oligo signals were detected in other PMCs from the same individual as Fig. 5. Figure S4. Tracing chromosome pairing at meiotic pachytene of S4 generation. Figure S5. Three representative PMCs with multivalents and one lagged C-bivalent at metaphase I in S4 generation. Figure S6. A heatmap depicting the frequency of different lagged bivalent numbers in the asynchronous meiotic PMCs of the S4 and S14 generation. [file 12870_2019_2060_MOESM1_ESM.pdf]

SUPPLEMENTAL MATERIAL

Figure:

Fig. S1

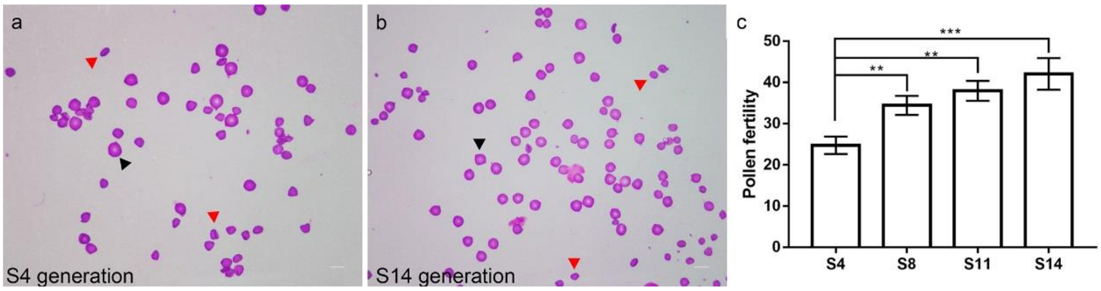

Fig. S1 Comparison of pollen viability in four generations of C. ×*hytivus* allotetraploid (S_4, S_8, S_{11} and S_{14}). (**a**-**b**) The plump pollen grains represent pollen fertility (black arrows), while unplump pollen grains represent pollen infertility (red arrows). (a) The mean value of S4 generation pollen fertility is 24.7%. (**b**) The mean value of S_{14} generation pollen fertility is 42.1%. (**c**) Pollen fertility of three generations (S_8, S_{11} and S_{14}) was significantly higher than S_4 generation. Error bars indicating the ±SD over five biological replicates. Asterisks indicating statistically significant differences at ** $p < 0.01$, *** $p < 0.001$.

Fig. S2

a

b

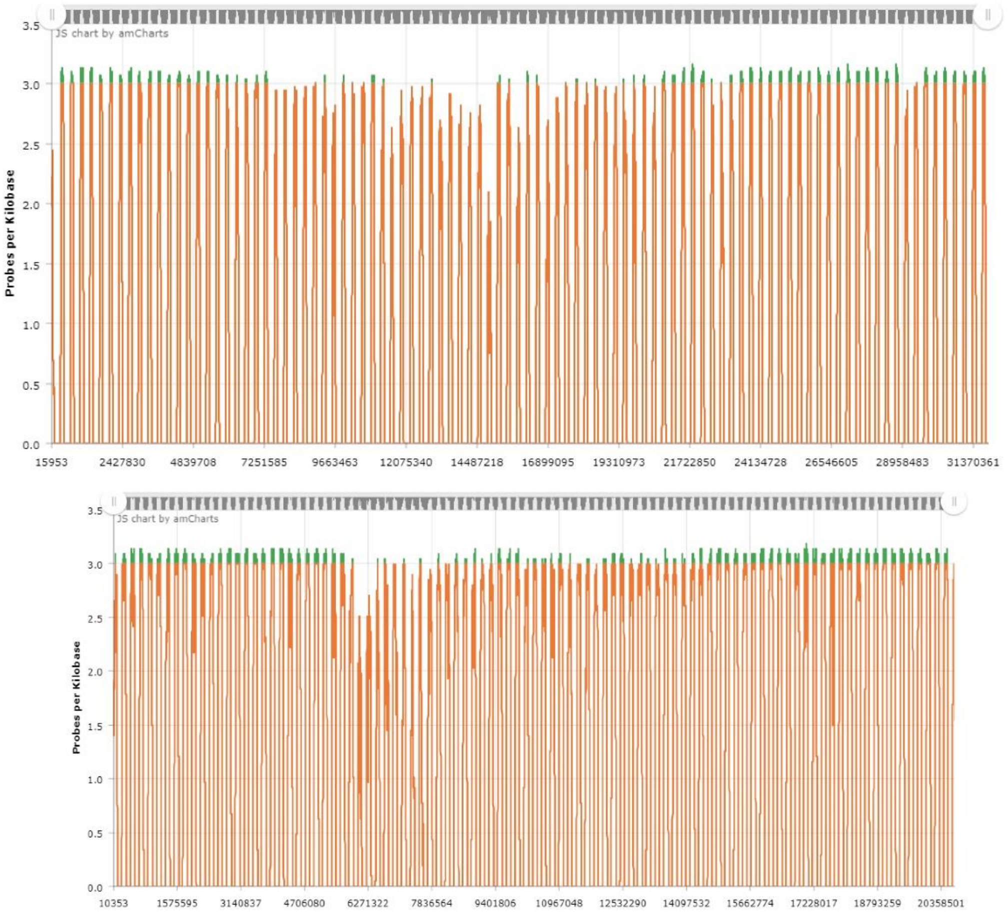

Fig. S2 Locations and density of 27,392 oligos along the sequence map of cucumber chromosomes. (**a**) The oligos information of cucumber chromosome 5. (**b**) The oligos information of cucumber chromosome 7. Oligos targeting 100kb per 300kb were chosen for chromosomes C5 and C7.

Fig. S3

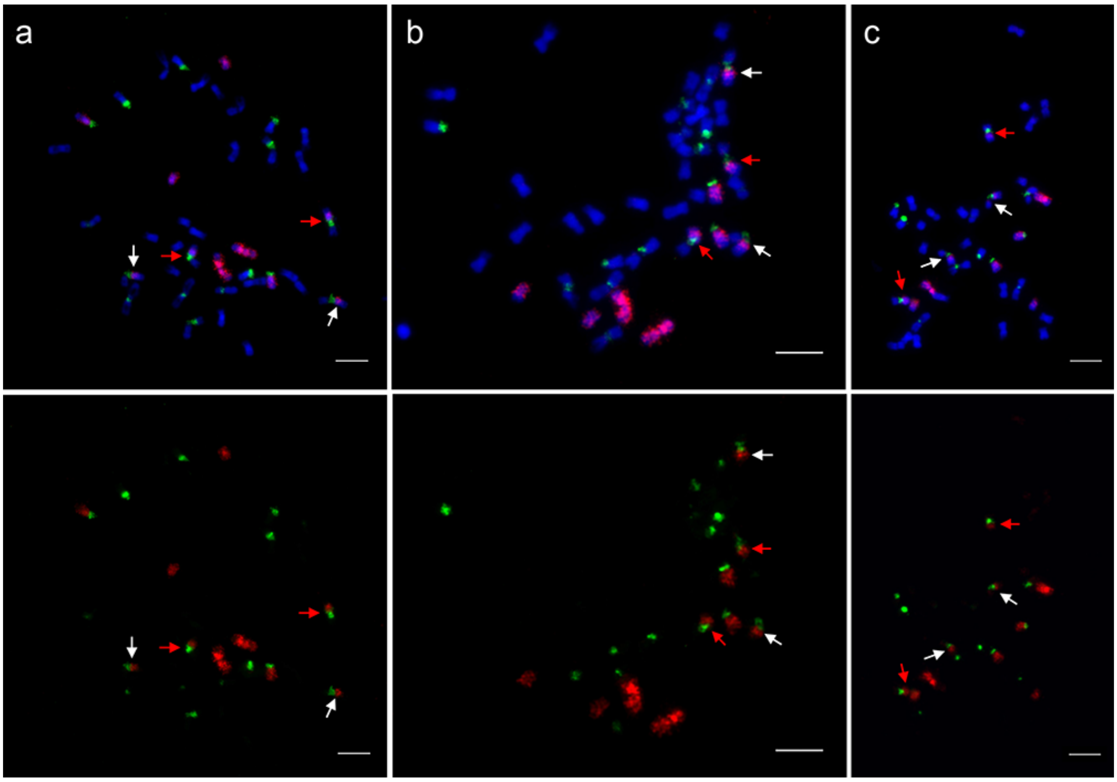

Fig. S3 Two translocations carrying C5-oligo signals were detected in other cells from same S_{14} individual as Fig. 5. (**a-c**) Red FISH signals from C-oligo probes; green FISH signals from 45S rDNA probes. Red arrows indicating one translocation anchored on C2; white arrows indicating another translocation anchored on C4.

Fig. S4

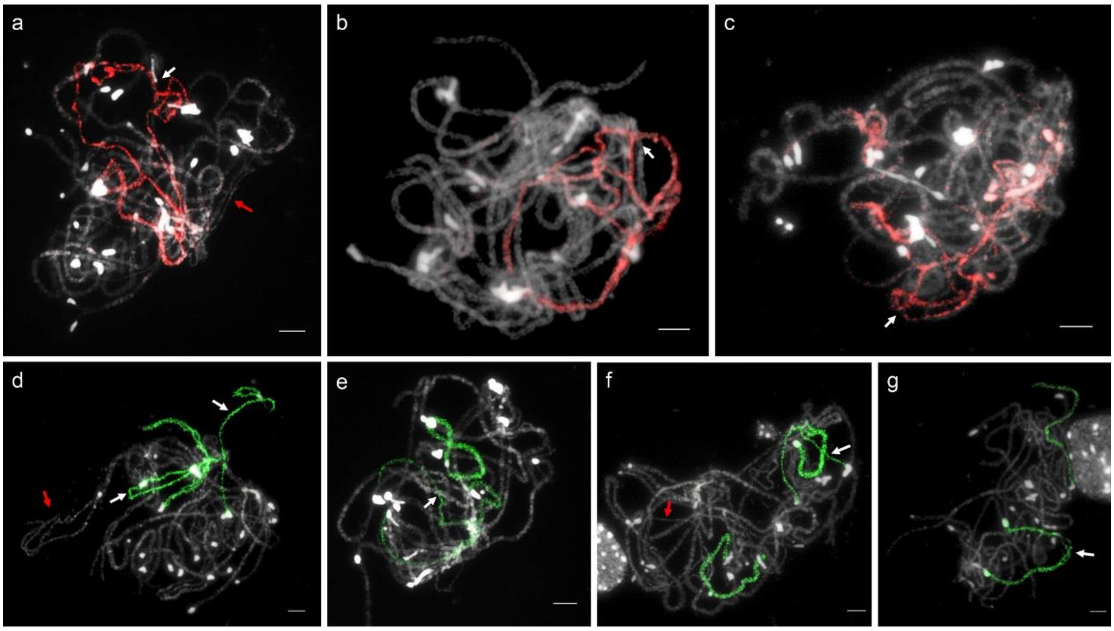

Fig. S4 Tracing chromosome pairing at meiotic pachytene of the S_4 generation. (**a-c**) Chromosome painting by C5-oligo probes (red). The chromosomes C5 and H9 or H10 were partially paired (white arrows). (**d-g**) Chromosome painting by C7-oligo probes (green). (**d**) An unpaired single chromosome string of C7 (white arrow). (**e**) An unpaired chromosome region of H1 (white arrow). (**f, g**) Unpaired chromosome region of C7 (white arrow). (**a, d, f**) Red arrows indicating unpaired single chromosome strings and/or chromosome regions. Bars = 5μm.

Fig. S5

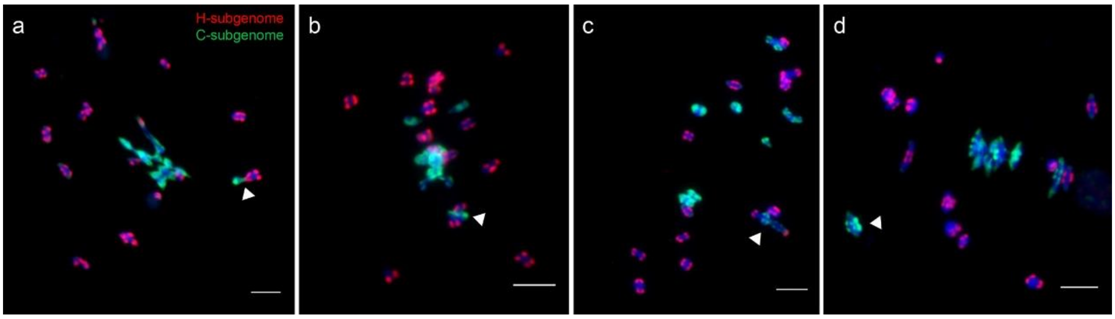

Fig. S5 Three representative PMCs with multivalents and one lagged C-bivalent at metaphase I in S_4 generation. (**a**) A HH-C trivalent (white arrow). (**b**) A H-CC-H tetravalent (white arrow). (**c**) A H-C-H trivalent (white arrow). (**d**) A C-bivalent did not reach the equatorial plate (white arrow). Bars = 5μm.

Fig. S6

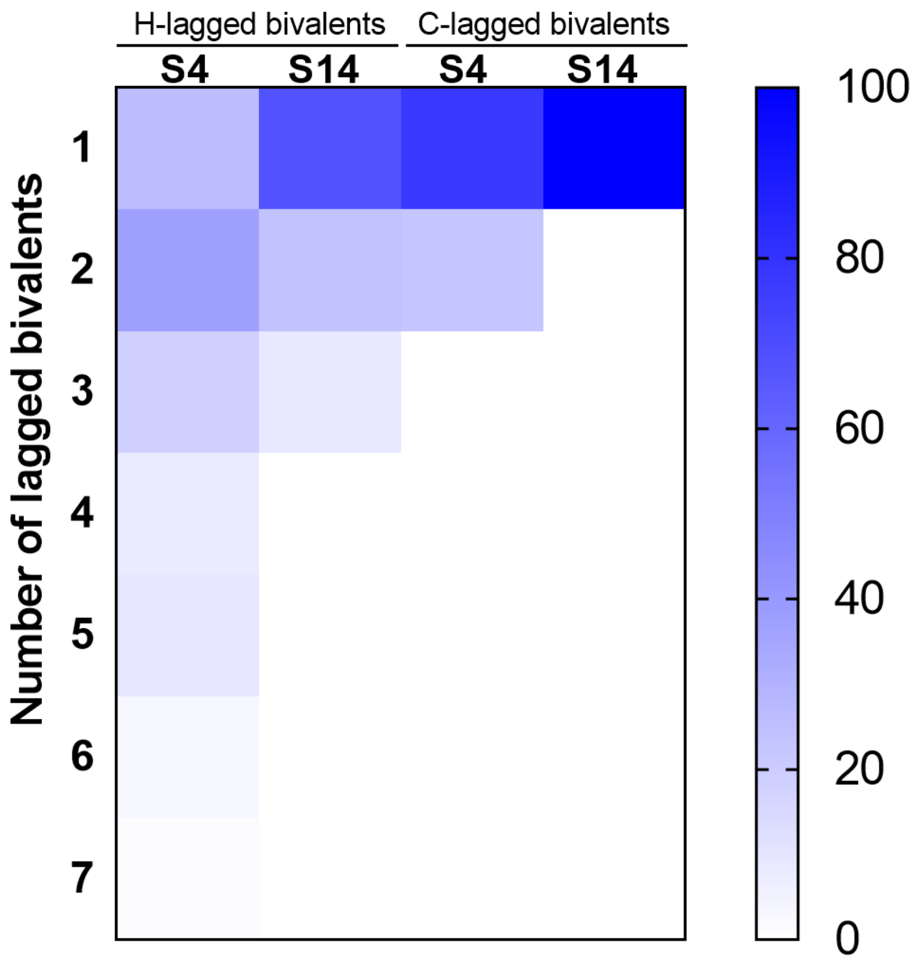

Fig. S6 A heatmap depicting the frequency of different lagged bivalent numbers in the asynchronous meiotic PMCs of S_4 and S_{14} generations. The y-axes denote the number of bivalents that did not reach the equatorial plate in each MI PMCs with asynchronous meiosis. The colored vertical bars refer to the frequencies of PMCs falling to each of different lagged bivalents in all asynchonous meiotic PMCs.
